# An updated checklist of the lichens of St. Eustatius, Netherlands Antilles

**DOI:** 10.3897/mycokeys.33.23911

**Published:** 2018-04-03

**Authors:** André Aptroot, Michael Stech

**Affiliations:** 1 ABL Herbarium, Gerrit van der Veenstraat 107, NL-3762 XK Soest, The Netherlands; 2 Naturalis Biodiversity Center, P.O. Box 9517, NL-2300 RA Leiden, The Netherlands; 3 Leiden University, Leiden, The Netherlands

**Keywords:** Biodiversity inventory, lichens, mtSSU, St. Eustatius

## Abstract

In the course of a multi-taxon biodiversity inventory for the island of St. Eustatius, lichens were collected from 11 plots representing different vegetation types. From these collections, 126 lichen species are reported, 54 of which are new reports for St. Eustatius. Most species could be identified to species level based on morphological and chemical characters. In a few cases, mtSSU DNA sequences were generated for a preliminary molecular identification and future phylogenetic studies. In total, 263 identified lichen species are currently known from St. Eustatius, as well as some additional genera with yet unidentified species and lichenicolous fungi.

## Introduction

Sint Eustatius is a small island (21 km^2^) in the northern Leeward Islands part of the West Indies. It is one of the six islands of the Netherlands Antilles and, since 2010, a special municipality of the Netherlands. Sint Eustatius is roughly divided into three parts, the Northern Hills, the urbanised central area (‘Cultuurvlakte’) and the southern part dominated by the steep dormant volcano The Quill (600 m elev.). Although the whole island of St. Eustatius has been heavily impacted by human activities, the northern and southern parts are nowadays designated as National Parks with varied vegetation types especially on the slopes of The Quill. The latter comprise, for example, thorny woodlands, deciduous to evergreen seasonal forests, dry evergreen forest, montane thickets and elfin woodland (Stoffers 1956). A re-classification of the vegetation of St. Eustatius, based on cluster analysis of sample plots, resulted in 13 vegetation types characterised by different combinations of individual vascular plant species (Freitas et al. 2014).

Just as in almost all other groups of organisms, lichens are most diverse in the tropics (Sipman and Aptroot 2001). On St. Eustatius, they are commonly present on various substrates, including tree bark and twigs, siliceous rock, limestone, dead wood and living leaves. Despite their abundance, the lichen flora of St. Eustatius is still incompletely known. The authors are aware of only nine publications citing in total 14 lichen species from St. Eustatius, *viz. Phyllopsora corallina* (Eschw.) Müll. Arg. and *P. parvifoliella* (Nyl.) Müll. Arg. (Brako 1991), *Anisomeridium excellens* (Müll. Arg.) R.C. Harris (Harris 1995), *Syncesia glyphysoides* (Fée) Tehler (Tehler 1997), *Pseudopyrenula subnudata* Müll. Arg. (Harris 1998, as P. diluta var. degenerans Vain.), *Stirtonia neotropica* Aptroot, described based on material from St. Eustatius and Costa Rica (Aptroot 2009), *Syncesia subintegra* Sipman, described based on material from St. Eustatius, as well as *S. farinacea* (Fée) Tehler, *S. glyphysoides* and *S. graphica* (Fr.) Tehler (Sipman 2009), *Roccella gracilis* Bory (Aptroot and Schumm 2011), *Dirina paradoxa* (Fée) Tehler (Tehler et al. 2013), as well as *Astrothelium bicolor* (Taylor) Aptroot & Lücking, *A. phlyctaena* (Fée) Aptroot & Lücking and *Pseudopyrenula subnudata* Müll. Arg. (Aptroot and Lücking 2016).

The main source of information about the lichen flora of St. Eustatius is the online portal ‘Plants and Lichens of St. Eustatius’ (Boom et al. 2009). It contains a list with 209 lichen species, based on specimens collected by H. Sipman and W.R. Buck in 2008, identified by H. Sipman and mostly hosted in B (some in NY). In addition, some identified specimens are present in various other herbaria, some of which can be searched online. For instance, the database of BR cites the following identified specimens from St. Eustatius, collected by R. Hensen in 1991 and identified by the first author: *Megalaria bengalensis* Jagadeesh Ram & Aptroot, *Porina mastoidea* (Ach.) Müll. Arg. and *Sticta xanthotropa* (Kremp.) D.J. Galloway.

In 2015, a plot-based, multi-taxon biodiversity inventory of St. Eustatius was carried out by Naturalis Biodiversity Center, the European Invertebrate Survey (EIS) and different Dutch non-governmental organisations, together with St. Eustatius National Parks Foundation (STENAPA) and students from different Dutch universities. Here, the lichen records of that inventory are reported and an updated checklist of the lichens known from St. Eustatius is presented.

## Materials and methods

As part of a multi-taxon inventory, lichens were collected on St. Eustatius from 11 plots (25 m × 25 m) in different main vegetation types according to Freitas et al. (2014). Two plots (H1, H2) were situated in the Northern Hills area, eight (M1−M5, M7−M9) on and around The Quill in the southern part of the island and one (U1) in the central urban area. Details concerning the location and vegetation characteristics of the plot locations and the multi-taxon sampling approach are described in van Andel et al. (2016). Lichens were collected using a knife or hammer and chisel and subsequently air-dried and stored in paper bags.

Specimens were observed and identified by the first author using an Olympus SZX7 stereomicroscope and an Olympus BX50 compound microscope with interference contrast, connected to a Nikon Coolpix digital camera. Sections were mounted in tap water, in which all measurements were also taken. The chemistry of selected specimens was investigated by thin-layer chromatography (Orange et al. 2001), using solvent A.

DNA analysis based on mitochondrial ribosomal small subunit (mtSSU) sequences was carried out for ten unidentifiable or provisionally identified specimens of good quality (indicated in Table 1). Although the nuclear ribosomal ITS region is the generally accepted fungal DNA barcode locus (Schoch et al. 2012), mtSSU was chosen since more mtSSU than ITS sequences have yet been published for several of the genera or families to which the respective specimens putatively belong.

Genomic DNA was extracted using the NucleoMag 96 Plant kit (Macherey-Nagel) on the KingFisher Flex Purification System (ThermoFisher Scientific). The mtSSU region was PCR-amplified following Zoller et al. (1999) in terms of primers (mrSSU1/mrSSU3R) and the PCR protocol. PCR products were purified and sequenced at BaseClear B.V. (www.baseclear.com) using the amplification primers. Sequences were assembled and edited using Geneious v8.1.8 (Biomatters Ltd.) and subjected to a BLAST search (Altschul et al. 1990) against the GenBank database (megablast; considering, where possible, BLAST results with E value of 0.0 and query cover >90 %). Sequences are available in GenBank under accession numbers MH028639−MH028646.

To compile an updated list of the lichens of St. Eustatius, literature and internet sources were exhaustively consulted for previous reports and previous collectors were contacted for additional information.

**Table 1.** Lichenised and lichenicolous fungi recorded in 11 plots on St. Eustatius. Species names in earlier publications are indicated in brackets. Author names are given in Table 2. Plots **H1** and **H2** are situated in the Northern Hills, **M2−M9** on and around the volcano The Quill in the southern part of the island and **U1** in the central urban area. Substrates occupied by each taxon are indicated per plot; **b**: bark, **k**: limestone, **l**: leaves, **r**: siliceous rock, **s**: soil, **w**: wood. Asterisks indicate first records for St. Eustatius (asterisks in brackets indicate additional taxa that are not yet identified to species level). Black dots (•) indicate specimens from which DNA was extracted.

Taxon	H1	H2	M1	M2	M3	M4	M5	M7	M8	M9	U1
(*)*Acanthothecis* sp. •				b							
*Alyxoria culmigena* (*Opegrapha herbarum*)	r						w		b		
*Alyxoria varia	b		b							b	b
*Amandinea multispora										b	
*Anisomeridium subprostans				b							
*Anisomeridium tamarindi*	b										
(*)*Anisomeridium* sp. corticate c. pycnidia •					b						
*Anisomeridium terminatum							b				
*Arthonia antillarum*		b				b					
*Arthonia caribaea*	b										
*Arthonia conferta*	b	b				b	b	b	b	b	
*Arthonia cyrtodes*				b							
*Arthonia minuta*					b					b	
*Arthonia parantillarum	b				b						
*Arthothelium macrothecum*				b	b						
*Bacidia medialis					b, r	r					
(*)*Bacidia* sp. apotheciate •							b, w				
(*)*Bacidia* sp. sorediate •					b						
*Bactrospora denticulata*	b				b	b			b	b	
*Bactrospora jenikii					b						
*Bogoriella annonacea							b		b		
*Brigantiaea leucoxantha				b							
*Buellia dejungens*		r				r	r	r		r	
*Buellia griseovirens							w		w		
*Buellia mamillana* (*Buellia glaziouana*)									r		
*Caloplaca leptozona*		r					r				
*Caloplaca obscurella							w				
*Coenogonium linkii*			b	b							
*Coenogonium saepincola							w				
*Coenogonium strigosum*				b							
*Coniocarpon cinnabarinum* (*Arthonia cinnabarina*)				b							
*Crespoa carneopruinata	b, r										
*Cresponea flava	r										
*Cryptothecia punctosorediata					b						
*Cryptothecia striata*				b	b, r						
(*)*Cryptothecia* sp. isidiate •			b								
(*)*Cryptothecia* sp. sterile •					b						
*Dactylospora saxatilis (lichenicolous on *Pertusaria praetervisa*)		r									
*Dichosporidium nigrocinctum*			b, r	b, r							
*Dictyomeridium amylosporum	b						b				
*Diorygma hieroglyphicum*				b							
*Diorygma poitaei*				b							
*Diorygma pruinosum				b							
*Endocarpon pallidulum		r				r		r, k	r, s	r	
*Enterographa pallidella*		b				b					
*Enterographa subserialis*									b		
*Flakea papillata*			b	b, r, w	b, r		r				
*Glyphis scyphulifera*									b		
*Graphis caesiella*					b						
*Graphis cincta					b						
*Graphis dendrogramma*				b			b				
*Graphis librata										b	
*Gyalolechia bassiae* (*Caloplaca bassiae*)						b	b, r				
*Hafellia curatellae										b	
*Hyperphyscia adglutinata*						b				b, r	b
*Lathagrium neglectum* (*Collema neglectum*)									b		
*Lecanora legalloana*	r				r	r	r	r			
*Lecanora leproplaca					b		b		b		
*Lecanora leprosa*							r				
*Lecanora prosecha*	r	r				r	r	r	r		
*Lecanora sulfurescens*	r										
*Lepraria finkii			b								
*Leprocollema nova-caledonianum						w					
*Letrouitia domingensis*				b							
*Malmidea piperis* (*Malcolmiella piperis*)				b							
*Malmidea psychotrioides				b							
*Malmidea vinosa* (*Malcolmiella vinosa*)			b								
*Mazosia carnea (Mazosia ocellata*)				b	b						
(*)*Melaspilea* sp. (lichenicolous on *Pyrenula dissimulans*)						b					
*Microtheliopsis uleana			l								
*Mycoporum eschweileri				b	b	b			b	b	
*Nyungwea anguinella* (*Enterographa anguinella*)					b						
*Opegrapha astraea						b	b				
*Opegrapha lithyrgiza	r										
*Opegrapha quintana					b						
(*)*Opegrapha* sp.								k			
*Peltula bolanderi*		r									
*Peltula obscurans*		r				r				r	
*Pertusaria coccopoda*						r		r			
*Pertusaria praetervisa*	r	r									
*Pertusaria texana									b		
*Pertusaria xanthodes*								b			
*Phaeographis crispata	b										
*Phaeographis dendritica*									b		
*Phyllopeltula corticola								b			
*Phyllopsora corallina*			b		b, r	r					
*Physcia atrostriata*				b							
*Physcia erumpens										b	
*Physcia integrata						b	b				
*Physcia sorediosa*						b					
*Porina conspersa				b, r	r						
*Porina epiphylla*			l								
*Porina internigrans*				b	b						
*Porina leptalea					r						
*Porina nucula*					b, r						
*Porina rubentior			l								
*Porina tetracerae*			b	b	r						
*Porina thaxteri			l								
(*)Psorotichia cf. americana							r				
*Pyrenopsis antillarum		r									
*Pyrenula adacta					b		b	b	b		
*Pyrenula breutelii* (*Pyrenula macularis*)							b			b	
*Pyrenula cocoes*	b				b	b	b		b		
*Pyrenula cruenta					b						
*Pyrenula dissimulans	b				b	b	b	b			
*Pyrenula nitidula*					b						
*Pyxine cocoes*						b, r	r	r	r		b
*Ramalina stoffersii				r							
*Rinodina antillarum						r					
*Rinodina colobinoides						b		b			
*Rinodina pyxinoides*	r	r				r	r		r	r	
Sarcographa cf. tricosa •					b						
*Squamulea subsoluta* (*Caloplaca subsoluta*)				r		r		r		r	
*Staurolemma dussii					b						
*Sticta xanthotropa			r	r							
*Stigmatochroma gerontoides									w		
(*)Stigmidium cf. schaereri								k			
*Strigula decipiens	r										
*Strigula phaea					b, r						
*Strigula smaragdula*			l								
*Syncesia decussans							b				
*Thelenella luridella						r		r	r		
(*)Thelidium cf. decipiens •								k			
(*)Verrucaria cf. dolosa •							r				
*Verrucaria nigrescens								r			
(*)Wetmoreana cf. appressa •		r								r	

## Results and discussion

In total, 126 lichen species (and one identifiable lichenicolous fungus) were found in 243 collections (Table 1). The vast majority (113 species) could be identified to species level based on morphological and chemical characters, even though no identification book exists for any region nearby. However, many species have been described from other islands in the Caribbean, which can be expected to have many species in common. These were often already described in the 19^th^ century and partly never studied again, but illustrations of their types are increasingly available. The authors also had access to various unpublished sources, such as the unpublished keys, descriptions and specimen citation (by H. Sipman) that was the basis of the internet checklist of St. Eustatius lichens and keys to the lichens from Puerto Rico (Harris 1989) and Guadeloupe (Øvstedal 2010), the latter with many illustrations of type and other specimens.

Somewhat to the authors’ surprise, as many as 54 (almost 50 %) of the identified species are new records for St. Eustatius. This includes mostly relatively common and widespread tropical or Neotropical species, but also some rare species, notably *Staurolemma dussii* (Vain.) P.M. Jørg. & Henssen, which was so far only known from its type from Guadeloupe. Furthermore, it is remarkable that *Cresponea flava* (Vain.) Egea & Torrente was found on siliceous rock. The presence of so many additional species within the limited surface area of the plots, totalling 6875 m^2^ (0.03% of the total island surface), suggests that the exploration of the lichen flora of St. Eustatius has not yet been exhaustive. However, no clearly undescribed species were found in the material and the number of species described based on material from St. Eustatius remains low with two, *viz. Stirtonia neotropica* (Aptroot 2009) and *Syncesia subintegra* (Sipman 2009).

Several specimens could not be identified with certainty in the present material but represent additional species (and in several cases additional genera). These are, for instance, Lichinaceae and Verrucariaceae, of which the taxonomy of the tropical taxa is incompletely known. Rather than describing them as new, they were listed with the name of the species that is morphologically most similar, preceded by “cf”. The BLAST results from the mtSSU sequences obtained from eight of these specimens in most cases allowed preliminary insights into their phylogenetic position.

The sequence of the *Anisomeridium* specimen with only conidia from St. Eustatius receives the highest BLAST hits with other representatives of the Monoblastiaceae in Nelsen et al. (2009, 2011), *viz. Anisomeridium ubianum* (Vain.) R.C. Harris, A. cf. willeyanum (R.C. Harris) R.C. Harris, *Megalotremis verrucosa* (Makhija & Patw.) Aptroot and *Trypetheliopsis kalbii* (Lücking & Sérus.) Aptroot. The low sequence identities of 86−93% clearly indicate that the St. Eustatius specimen belongs to another species in that family, but too few mtSSU sequences are yet available for a more precise molecular identification.

In the Graphidaceae, the top five BLAST hits for the specimen of *Acanthothecis* sp. were all with *Acanthothecis peplophora* (M. Wirth & Hale) E. Tripp & Lendemer specimens (97% identity), whereas the identity with the sequence of the type species of *Acanthothecis*, *A. hololeucoides* (Nyl.) Staiger & Kalb, was only 89%. The specimen from St. Eustatius thus most probably does not belong to *Acanthothecis* s.str., but may represent a species of ‘*Acanthothecis* 2’ in the *Carbacanthographis* clade (cf. Rivas Plata et al. 2013, Medeiros et al. 2017). The Sarcographa cf. tricosa specimen received BLAST hits of 97% identity with *Sarcographina glyphiza* (Nyl.) Kr.P. Singh & D.D. Awasthi and *Pallidogramme chlorocarpoides* (Nyl.) Staiger, Kalb & Lücking, both situated in the Graphioideae tribe Graphidae p.p. clade of Rivas Plata et al. (2013). However, another GenBank sequence of *P. chlorocarpoides*, as well as several species of other genera of same clade, were 96 % identical, including the single other specimen of *S. tricosa* in GenBank (but not the species of the *Sarcographa* s.str. clade *sensu* Rivas Plata et al. 2013). The identity of the St. Eustatius specimen thus remains ambiguous based on the presently available mtSSU sequence data.

Both the apotheciate and sorediate *Bacidia* specimens are closest to sequences of species of the *Toninia*-*Bacidia* p.p. clade in Miadlikowska et al. (2014), the former to *Toninia sedifolia* (Scop.) Timdal (94 % identity) and the latter to *Bacidia californica* S. Ekman and *B. phacodes* Körb. (88−89 % identity), respectively. Consequently, they do not belong to *Bacidia* s.str., which forms a separate clade (including the type species, *B. rosella* (Pers.) De Not.) in Miadlikowska et al. (2014).

In the Verrucariaceae, *Verrucaria* was resolved as polyphyletic and *Thelidium* mixed with *Polyblastia*, *Staurothele* p.p. and *Verrucaria* p.p. (*Polyblastia* clade) in molecular phylogenetic reconstructions (Gueidan et al. 2007, Muggia et al. 2010, Thüs et al. 2011). The sequence of the Thelidium cf. decipiens specimen from St. Eustatius, however, is closest to the *Catapyrenium*-*Placidiopsis*-*Verrucaria* p.p. (*V. caerulea* DC., *V. praetermissa* (Trevis.) Anzi) clade (Muggia et al. 2010) with sequence identities of 96−97 %. The placement of the Verrucaria cf. dolosa specimen is more difficult to assess, since its sequence shows lower similarities of 92−94 % to representatives of different Verrucariceae genera, such as *Agonimia*, *Bagliettoa* and *Verrucaria* spp.

Finally, the mtSSU sequence of the Wetmoreana cf. appressa specimen from St. Eustatius is difficult to interpret, since it matches more closely with sequences of the Xanthorioideae (sequence identity 97−99 %) than with Teloschistoideae, in which *Wetmoreana* is placed (e.g. Arup et al. 2013).

The lichen flora of St. Eustatius can be characterised as lowland, relatively dry Caribbean. As can be seen from Table 1, most species were found on one substratum type, but some are less specialised. Also, there is a marked difference between the lichens of the different plots and the three main areas on St. Eustatius (Northern Hills, central urban area, The Quill). However, the authors refrain from performing statistical comparisons of the lichen diversity between plots, since the number of plots per main area differs and is still low and the sampling strategy was devised by specialists of other organism groups. Nevertheless, the lichen data will be useful for an island-wide, plot-based comparison of diversity amongst all organism groups sampled during the 2015 inventory.

In Table 2, an updated checklist is presented of the lichens of St. Eustatius, citing only identified species, but based on all available sources and with their taxonomy (nomenclature and sometimes species concept) updated where necessary. According to this list, a total of 263 species are currently known from St. Eustatius. As a side effect of revising the existing records, one record becomes questionable, *viz. Myriostigma candidum* Kremp., which is not known from the Neotropics. It is intended to continue the exploration of the lichens of this island in the near future.

**Table 2.** Updated checklist of the lichens of St. Eustatius.

Species	References
**Lichens**	
*Acarospora chrysops* (Tuck.) H.Magn.	Boom et al. (2009) as *Acarospora dissipata* H.Magn.
*Alyxoria culmigena* (Lib.) Ertz	Boom et al. (2009) as *Opegrapha herbarum* Mont., present study
*Alyxoria ochrocheila* (Nyl.) Ertz & Tehler	Boom et al. (2009) as *Opegrapha ochrocheila* Nyl.
*Alyxoria varia* (Pers.) Ertz & Tehler	present study
*Amandinea efflorescens* (Müll. Arg.) Marbach	Boom et al. (2009)
*Amandinea multispora* (Kalb & Vězda) Marbach	present study
*Amandinea prospersa* (Nyl.) Elix & H. Mayhofer	Boom et al. (2009) as *Buellia prospersa* (Nyl.) Riddle
*Anisomeridium americanum* (A.Massal.) R.C. Harris	Boom et al. (2009)
*Anisomeridium excellens* (Müll. Arg.) R.C. Harris	Boom et al. (2009), Harris (1995)
*Anisomeridium subprostans* (Nyl.) R.C. Harris	present study
*Anisomeridium tamarindi* (Fée) R.C. Harris	Boom et al. (2009), present study
*Anisomeridium terminatum* (Nyl.) R.C. Harris	present study
*Anisomeridium tuckerae* (R.C. Harris) R.C. Harris	Boom et al. (2009)
*Arthonia antillarum* (Fée) Nyl.	Boom et al. (2009), present study
*Arthonia caribaea* (Ach.) A. Massal.	Boom et al. (2009), present study
*Arthonia conferta* (Fée) Nyl.	Boom et al. (2009), present study
*Arthonia cyanea* Müll. Arg.	Boom et al. (2009)
*Arthonia cyrtodes* Nyl.	Boom et al. (2009), present study
*Arthonia minuta* Vain.	Boom et al. (2009), present study
*Arthonia parantillarum* Aptroot	present study
*Arthothelium macrothecum* (Fée) A. Massal.	Boom et al. (2009), present study
*Astrothelium bicolor* (Taylor) Aptroot & Lücking	Boom et al. (2009) as *Trypethelium nitidiusculum* (Nyl.) R.C. Harris, Aptroot & Lücking (2016)
*Astrothelium phlyctaena* (Fée) Aptroot & Lücking	Boom et al. (2009) as *Trypethelium ochroleucum* (Eschw.) Nyl., Aptroot & Lücking (2016)
*Bacidia medialis* (Tuck.) Zahlbr.	present study
*Bactrospora denticulata* (Vain.) Egea & Torrente	Boom et al. (2009), present study
*Bactrospora jenikii* (Vězda) Egea & Torrente	present study
*Bactrospora myriadea* (Fée) Egea & Torrente	Boom et al. (2009)
*Baculifera intermedioides* Marbach	Boom et al. (2009)
*Blastenia brittonii* Zahlbr.	Boom et al. (2009) as *Caloplaca brittonii* (Zahlbr.) ined.
*Bogoriella annonacea* (Müll. Arg.) Aptroot & Lücking	present study
*Brigantiaea leucoxantha* (Spreng.) R. Sant. & Hafellner	present study
*Brownliella cinnabarina* (Ach.) S.Y. Kondr., Kärnefelt, A. Thell, Elix, J.Kim, A.S.Kondr. & J.-S.Hur	Boom et al. (2009) as *Caloplaca cinnabarina* (Ach.) Zahlbr.
*Buellia boergesenii* Imshaug	Boom et al. (2009)
*Buellia dejungens* (Nyl.) Vain.	Boom et al. (2009), present study
*Buellia griseovirens* (Turner & Borrer ex Sm.) Almb.	present study
*Buellia mamillana* (Tuck.) W.A. Weber	Boom et al. (2009) as *Buellia glaziouana* (Kremp.) Müll. Arg., present study
*Buellia posthabita* (Nyl.) Zahlbr.	Boom et al. (2009)
*Bulbothrix scortella* (Nyl.) Hale	Boom et al. (2009)
*Bulbothrix suffixa* (Stirt.) Hale	Boom et al. (2009)
*Byssoloma leucoblepharum* (Nyl.) Vain.	Boom et al. (2009)
*Caloplaca diplacia* (Ach.) Riddle	Boom et al. (2009)
*Caloplaca leptozona* (Nyl.) Zahlbr.	Boom et al. (2009), present study
*Caloplaca obscurella* (J. Lahm) Th. Fr.	present study
*Canoparmelia martinicana* (Nyl.) Elix & Hale	Boom et al. (2009)
*Carbacanthographis triphoroides* (M. Wirth & Hale) Lücking	Boom et al. (2009)
*Chapsa cinchonarum* (Fée) Frisch	Boom et al. (2009)
*Chrysothrix xanthina* (Vain.) Kalb	Boom et al. (2009)
*Cladonia corymbites* Nyl.	Boom et al. (2009)
*Cladonia didyma* (Fée) Vain.	Boom et al. (2009)
*Cladonia subradiata* (Vain.) Sandst.	Boom et al. (2009)
*Coccocarpia palmicola* (Spreng.) Arv. & D.J. Galloway	Boom et al. (2009)
*Coccocarpia pellita* (Ach.) Müll. Arg.	Boom et al. (2009)
*Coenogonium interpositum* Nyl.	Boom et al. (2009)
*Coenogonium leprieurii* (Mont.) Nyl.	Boom et al. (2009)
*Coenogonium linkii* Ehrenb.	Boom et al. (2009), present study
*Coenogonium saepincola* Aptroot, Sipman & Lücking	present study
*Coenogonium strigosum* Rivas Plata, Lücking & Chaves	Boom et al. (2009), present study
*Coenogonium subdilutum* (Malme) Lücking, Aptroot & Sipman	Boom et al. (2009)
*Coniocarpon cinnabarinum* DC.	Boom et al. (2009) as *Arthonia cinnabarina* (DC.) Wallr., present study
*Cratiria lauricassiae* (Fée) Marbach	Boom et al. (2009)
*Crespoa carneopruinata* (Zahlbr.) Lendemer & B.P. Hodk.	present study
*Cresponea flava* (Vain.) Egea & Torrente	present study
*Cresponea leprieurii* (Mont.) Egea & Torrente	Boom et al. (2009)
*Cresponea proximata* (Nyl.) Egea & Torrente	Boom et al. (2009)
*Cryptolechia carneolutea* (Tuck.) A. Massal.	Boom et al. (2009)
*Cryptothecia megalocarpa* (Müll. Arg.) R. Sant.	Boom et al. (2009)
*Cryptothecia punctosorediata* Sparrius	present study
*Cryptothecia striata* G. Thor	Boom et al. (2009), present study
*Dichosporidium nigrocinctum* (Ehrenb.) G. Thor	Boom et al. (2009), present study
*Dictyomeridium amylosporum* (Vain.) Aptroot, M.P. Nelsen & Lücking	present study
*Diorygma hieroglyphicum* (Pers.) Staiger & Kalb	Boom et al. (2009), present study
*Diorygma poitiaei* (Fée) Kalb, Staiger & Elix	Boom et al. (2009), present study
*Diorygma pruinosum* (Eschw.) Kalb, Staiger & Elix	present study
*Diploschistes actinostomus* (Ach.) Zahlbr.	Boom et al. (2009)
*Diploschistes aeneus* (Müll. Arg.) Lumbsch	Boom et al. (2009)
*Diploschistes prominens* (Vain.) Lumbsch	Boom et al. (2009)
*Dirina paradoxa* (Fée) Tehler	Boom et al. (2009) as Dirina approximata subsp. hioramii (B. de Lesd.) Tehler, Tehler et al. (2013)
*Dirinaria aegialita* (Ach.) B.J. Moore	Boom et al. (2009)
*Endocarpon pallidulum* (Nyl.) Nyl.	present study
*Enterographa compunctula* (Nyl.) Redinger	Boom et al. (2009)
*Enterographa multilocularis* (Müll. Arg.) Sparrius	Boom et al. (2009)
*Enterographa pallidella* (Nyl.) Redinger	Boom et al. (2009), present study
*Enterographa perez-higaredae* Herrera-Camp. & Lücking	Boom et al. (2009)
*Enterographa sipmanii* Sparrius	Boom et al. (2009)
*Enterographa subserialis* (Nyl.) Redinger	Boom et al. (2009), present study
*Eremothecella microcephalica* Sipman	Boom et al. (2009)
*Fellhanera santessonii* Barillas & Lücking	Boom et al. (2009)
*Fissurina dumastii* Fée	Boom et al. (2009)
*Flakea papillata* O.E. Erikss.	Boom et al. (2009), present study
*Glyphis cicatricosa* Ach.	Boom et al. (2009)
*Glyphis scyphulifera* (Ach.) Staiger	Boom et al. (2009), present study
*Graphis caesiella* Vain.	Boom et al. (2009), present study
*Graphis chondroplaca* (Redinger) Lücking	Boom et al. (2009)
*Graphis cincta* (Pers.) Aptroot	present study
*Graphis dendrogramma* Nyl.	Boom et al. (2009), present study
*Graphis furcata* Fée	Boom et al. (2009)
*Graphis glaucescens* Fée	Boom et al. (2009)
*Graphis librata* C. Knight	present study
*Graphis lineola* Ach.	Boom et al. (2009)
*Graphis tenella* Ach.	Boom et al. (2009)
*Graphis tenellula* Vain.	Boom et al. (2009)
*Gyalectidium filicinum* Müll. Arg.	Boom et al. (2009)
*Gyalolechia bassiae* (Ach.) Søchting, Frödén & Arup ex Ahti	Boom et al. (2009) as *Caloplaca bassiae* (Ach.) Zahlbr., present study
*Hafellia bahiana* (Malme) Sheard	Boom et al. (2009)
*Hafellia curatellae* (Malme) Marbach	present study
*Herpothallon aurantiacoflavum* (B. de Lesd.) Aptroot, Lücking & G.Thor	Boom et al. (2009)
*Heterodermia albicans* (Pers.) Swinscow & Krog	Boom et al. (2009)
*Heterodermia galactophylla* (Tuck.) W.L. Culb.	Boom et al. (2009)
*Heterodermia lutescens* (Kurok.) Follmann	Boom et al. (2009)
*Heterodermia obscurata* (Nyl.) Trevis.	Boom et al. (2009)
*Heterodermia squamulosa* (Degel.) W.L. Culb.	Boom et al. (2009)
*Heterodermia verrucifera* (Kurok.) W.A. Weber	Boom et al. (2009)
*Hyperphyscia adglutinata* (Flörke) H. Mayrhofer & Poelt	Boom et al. (2009), present study
*Hyperphyscia minor* (Fée) D.D. Awasthi	Boom et al. (2009)
*Lathagrium neglectum* (Degel.) Otálora, P.M. Jørg. & Wedin	Boom et al. (2009) as *Collema neglectum* Degel., present study
*Lecanactis epileuca* (Nyl.) Tehler	Boom et al. (2009)
*Lecanora galactiniza* Nyl.	Boom et al. (2009)
*Lecanora legalloana* Elix & Øvstedal	Boom et al. (2009), present study
*Lecanora leproplaca* Zahlbr.	present study
*Lecanora leprosa* Fée	Boom et al. (2009), present study
*Lecanora prosecha* Ach.	Boom et al. (2009), present study
*Lecanora sulfurescens* Fée	Boom et al. (2009), present study
*Leiorreuma exaltatum* (Mont. & Bosch) Staiger	Boom et al. (2009)
*Lepraria finkii* (B. de Lesd.) R.C. Harris	present study
*Leprocollema novacaledonianum* A.L. Sm.	present study
*Leptogium austroamericanum* (Malme) C.W. Dodge	Boom et al. (2009)
*Leptogium azureum* (Sw.) Mont.	Boom et al. (2009)
*Leptogium cyanescens* (Rabenh.) Körb.	Boom et al. (2009)
*Leptogium marginellum* (Sw.) Gray	Boom et al. (2009)
*Letrouitia domingensis* (Pers.) Hafellner & Bellem.	Boom et al. (2009), present study
*Leucodecton bisporum* (Nyl.) Sipman & Lücking	Boom et al. (2009)
*Leucodecton compunctum* (Ach.) A. Massal.	Boom et al. (2009)
*Loflammia gabrielis* (Müll. Arg.) Vezda	Boom et al. (2009)
*Malmidea piperis* (Spreng.) Kalb, Rivas Plata & Lumbsch	Boom et al. (2009) as *Malcolmiella piperis* (Spreng.) Kalb & Lücking, present study
*Malmidea psychotrioides* (Kalb & Lücking) Kalb, Rivas Plata & Lumbsch	present study
*Malmidea vinosa* (Eschw.) Kalb, Rivas Plata & Lumbsch	Boom et al. (2009) as *Malcolmiella vinosa* (Eschw.) Kalb & Lücking, present study
*Mazosia carnea* (Eckfelt) Aptroot & M. Cáceres	Boom et al. (2009) as *Mazosia ocellata* (Nyl.) R.C. Harris, present study
*Mazosia phyllosema* (Nyl.) Zahlbr.	Boom et al. (2009)
*Megalaria bengalensis* Jagadeesh Ram & Aptroot	Hensen (BR)
*Melanotrema meiospermum* (Nyl.) Frisch	Boom et al. (2009)
*Microtheliopsis uleana* Müll. Arg.	present study
*Mycoporum eschweileri* (Müll. Arg.) R.C. Harris	present study
*Myriostigma candidum* Kremp.	Boom et al. (2009) as *Cryptothecia candida* (Kremp.) R. Sant.: incorrect report
*Myriotrema myriotremoides* (Nyl.) Hale	Boom et al. (2009)
*Myriotrema olivaceum* Fée	Boom et al. (2009)
*Nyungwea anguinella* (Nyl.) Aptroot	Boom et al. (2009) as *Enterographa anguinella* (Nyl.) Redinger, present study
*Ocellularia depressa* (Mont.) Hale	Boom et al. (2009)
*Ocellularia interposita* (Nyl.) Hale	Boom et al. (2009)
*Ocellularia terebrata* (Ach.) Müll. Arg.	Boom et al. (2009)
*Opegrapha astraea* Tuck.	present study
*Opegrapha lithyrgiza* Vain.	present study
*Opegrapha quintana* Redinger	present study
*Pannaria prolificans* Vain.	Boom et al. (2009)
*Parmotrema crinitum* (Ach.) M. Choisy	Boom et al. (2009)
*Parmotrema endosulphureum* (Hillmann) Hale	Boom et al. (2009)
*Parmotrema praesorediosum* (Nyl.) Hale	Boom et al. (2009)
*Parmotrema tinctorum* (Nyl.) Hale	Boom et al. (2009)
*Parmotrema ultralucens* (Krog) Hale	Boom et al. (2009)
*Peltula bolanderi* (Tuck.) Wetmore	Boom et al. (2009), present study
*Peltula obscurans* (Nyl.) Gyeln.	Boom et al. (2009), present study
*Pertusaria coccopoda* Vain.	Boom et al. (2009), present study
*Pertusaria leioplacella* Nyl.	Boom et al. (2009)
*Pertusaria praetervisa* Vain.	Boom et al. (2009), present study
*Pertusaria texana* Müll. Arg.	present study
*Pertusaria xanthodes* Müll. Arg.	Boom et al. (2009), present study
*Phaeographis crispata* Kalb & Matthes-Leicht	present study
*Phaeographis dendritica* (Ach.) Müll. Arg.	Boom et al. (2009), present study
*Phaeographis intricans* (Nyl.) Staiger	Boom et al. (2009)
*Phaeographis scalpturata* (Ach.) Staiger	Boom et al. (2009)
*Phyllopeltula corticola* (Büdel & R. Sant.) Kalb	present study
*Phyllopsora chlorophaea* (Müll. Arg.) Zahlbr.	Boom et al. (2009)
*Phyllopsora corallina* (Eschw.) Müll. Arg.	Brako (1991), Boom et al. (2009), present study
*Phyllopsora glaucescens* Timdal	Boom et al. (2009)
*Phyllopsora parvifoliella* (Nyl.) Müll. Arg.	Brako (1991), Boom et al. (2009)
*Physcia atrostriata* Moberg	Boom et al. (2009), present study
*Physcia crispa* Nyl.	Boom et al. (2009)
*Physcia erumpens* Moberg	present study
*Physcia integrata* Moberg	present study
*Physcia sinuosa* Moberg	Boom et al. (2009)
*Physcia sorediosa* (Vain.) Lynge	Boom et al. (2009), present study
*Physcia tenuis* Moberg	Boom et al. (2009)
*Platythecium colliculosum* (Mont.) Staiger	Boom et al. (2009)
*Platythecium leiogramma* (Nyl.) Staiger	Boom et al. (2009)
*Polymeridium quinqueseptatum* (Nyl.) R.C. Harris	Boom et al. (2009)
*Porina conspersa* Malme	present study
*Porina epiphylla* (Fée) Fée	Boom et al. (2009), present study
*Porina internigrans* (Nyl.) Müll. Arg.	Boom et al. (2009), present study
*Porina leptalea* (Durieu & Mont.) A.L. Sm.	present study
*Porina mastoidea* (Ach.) Müll. Arg.	Hensen (BR), Boom et al. (2009)
*Porina nitidula* Müll. Arg.	Boom et al. (2009)
*Porina nucula* Ach.	Boom et al. (2009), present study
*Porina octomera* (Müll. Arg.) F.Schill.	Boom et al. (2009)
*Porina rubentior* (Stirt.) Müll. Arg.	present study
*Porina tetracerae* (Ach.) Müll. Arg.	Boom et al. (2009), present study
*Porina thaxteri* R. Sant.	present study
*Pseudochapsa dilatata* (Müll. Arg.) Parnmen, Lücking & Lumbsch	Boom et al. (2009) as *Chapsa dilatata* (Müll. Arg.) Kalb
*Pseudopyrenula subgregaria* Müll. Arg.	Boom et al. (2009)
*Pseudopyrenula subnudata* Müll. Arg.	Harris (1998) as Pseudopyrenula diluta (Fée) Müll. Arg. var. degenerans Vain, Boom et al. (2009) as *Pseudopyrenula diluta*, Aptroot & Lücking (2016)
*Pyrenopsis antillarum* Vain.	present study
*Pyrenula adacta* Fée	present study
*Pyrenula astroidea* (Fée) R.C. Harris	Boom et al. (2009)
*Pyrenula bahiana* Malme	Boom et al. (2009) as *Pyrenula concatervans* (Nyl.) R.C. Harris
*Pyrenula breutelii* (Müll. Arg.) Aptroot	Boom et al. (2009) as *Pyrenula macularis* (Zahlbr.) R.C. Harris, present study
*Pyrenula cinerea* Zahlbr.	Boom et al. (2009)
*Pyrenula cocoes* Müll. Arg.	Boom et al. (2009), present study
*Pyrenula confinis* (Nyl.) R.C. Harris	Boom et al. (2009)
*Pyrenula cruenta* (Mont.) Vain.	present study
*Pyrenula dissimulans* (Müll. Arg.) R.C. Harris	present study
*Pyrenula duplicans* (Nyl.) Aptroot	Boom et al. (2009)
*Pyrenula leucostoma* Ach.	Boom et al. (2009)
*Pyrenula mamillana* (Ach.) Trevis.	Boom et al. (2009) as *Pyrenula xyloides* (Eschw.) Müll. Arg.
*Pyrenula massariospora* (Starbäck) R.C. Harris	Boom et al. (2009)
*Pyrenula microtheca* R.C. Harris	Boom et al. (2009)
*Pyrenula nitidula* (Bres.) R.C. Harris	Boom et al. (2009), present study
*Pyrenula septicollaris* (Eschw.) R.C. Harris	Boom et al. (2009)
*Pyxine cocoes* (Sw.) Nyl.	Boom et al. (2009), present study
*Ramalina anceps* Nyl.	Boom et al. (2009)
*Ramalina complanata* (Sw.) Ach.	Boom et al. (2009)
*Ramalina dendroides* (Nyl.) Nyl.	Boom et al. (2009)
*Ramalina furcellata* (Ach.) Zahlbr.	Boom et al. (2009)
*Ramalina stoffersii* Sipman	present study
*Rinodina antillarum* Vain.	present study
*Rinodina colobinoides* (Nyl.) Müll. Arg.	present study
*Rinodina pyxinoides* Vain.	Boom et al. (2009), present study
*Roccella gracilis* Bory	Boom et al. (2009), Aptroot and Schumm (2011)
*Roccellographa circumscripta* (Leight.) Ertz & Tehler	Boom et al. (2009) as *Peterjamesia circumscripta* (Taylor) D. Hawksw.
*Sarcographa heteroclita* (Mont.) Zahlbr.	Boom et al. (2009)
*Sarcographa labyrinthica* (Ach.) Müll. Arg.	Boom et al. (2009)
*Sarcographa ramificans* (Kremp.) Staiger	Boom et al. (2009)
*Sarcographa tricosa* (Ach.) Müll. Arg.	Boom et al. (2009), present study
*Sclerophyton elegans* Eschw.	Boom et al. (2009)
*Sclerophyton trinidadense* Sparrius	Boom et al. (2009)
*Sporopodium phyllocharis* (Mont.) A. Massal.	Boom et al. (2009)
*Squamulea subsoluta* (Nyl.) Arup, Søchting & Frödén	Boom et al. (2009) as *Caloplaca subsoluta* (Nyl.) Zahlbr., present study
*Staurolemma dussii* (Vain.) P.M. Jørg. & Henssen	present study
*Stegobolus auberianus* (Mont.) Frisch & Kalb	Boom et al. (2009)
*Stegobolus granulosus* (Tuck.) Frisch	Boom et al. (2009)
*Stegobolus subcavatus* (Nyl.) Frisch	Boom et al. (2009)
*Sticta xanthotropa* (Kremp.) D.J. Galloway	Hensen (BR), present study
*Stigmatochroma gerontoides* (Stirt.) Marbach	present study
*Stirtonia neotropica* Aptroot	Aptroot (2009)
*Strigula decipiens* (Malme) P.M. McCarthy	present study
*Strigula macrospora* Vain.	Boom et al. (2009)
*Strigula nemathora* Mont.	Boom et al. (2009)
*Strigula obducta* (Müll. Arg.) R.C. Harris	Boom et al. (2009)
*Strigula phaea* (Ach.) R.C. Harris	present study
*Strigula smaragdula* Fr.	Boom et al. (2009), present study
*Synalissa lichinella* Vain.	Boom et al. (2009)
*Syncesia decussans* (Nyl.) Tehler	present study
*Syncesia farinacea* (Fée) Tehler	Boom et al. (2009), Sipman (2009)
*Syncesia glyphysoides* (Fée) Tehler	Tehler (1997), Boom et al. (2009), Sipman (2009)
*Syncesia graphica* (Fr.) Tehler	Boom et al. (2009), Sipman (2009)
*Syncesia subintegra* Sipman	Boom et al. (2009), Sipman (2009)
*Teloschistes flavicans* (Sw.) Norman	Boom et al. (2009) as Teloschistes flavicans var. crocea (Ach.) Müll. Arg.
*Thalloloma hypoleptum* (Nyl.) Staiger	Boom et al. (2009)
*Thelenella luridella* (Nyl.) H. Mayrhofer	present study
*Thelotrema porinoides* Mont. & Bosch	Boom et al. (2009)
*Toninia submexicana* B. de Lesd.	Boom et al. (2009)
*Trapelia coarctata* (Sm.) M. Choisy	Boom et al. (2009)
*Usnea baileyi* (Stirt.) Zahlbr.	Boom et al. (2009)
*Varicellaria velata* (Turner) I. Schmitt & Lumbsch	Boom et al. (2009)
*Verrucaria nigrescens* Pers.	present study
*Xanthoparmelia succedans* Elix & J. Johnst.	Boom et al. (2009)
**Lichenicolous fungus**	
*Dactylospora saxatilis* (Schaer.) Hafellner (lichenicolous on *Pertusaria praetervisa*)	present study
**Additional genera (species uncertain)**	
*Acanthothecis* sp.	present study
*Bacidina* sp.	present study
*Melaspilea* sp. (lichenicolous fungus)	present study
*Psorotichia* sp.	present study
*Stigmidium* sp. (lichenicolous fungus)	present study
*Thelidium* sp.	present study
*Wetmoreana* sp.	present study
